# Skin and stinger bacterial communities in two critically endangered rays from the South Atlantic in natural and aquarium settings

**DOI:** 10.1002/mbo3.1141

**Published:** 2020-11-23

**Authors:** Fernanda Gonçalves e Silva, Henrique Fragoso dos Santos, Deborah Catharine de Assis Leite, Daniela Silva Lutfi, Marcelo Vianna, Alexandre Soares Rosado

**Affiliations:** ^1^ BioTecPesca‐Laboratory of Biology and Fisheries Technology—Institute of Biology Federal University of Rio de Janeiro (UFRJ) Rio de Janeiro Brazil; ^2^ LEMM, Laboratory of Molecular Microbial Ecology Institute of Microbiology Paulo de Góes Federal University of Rio de Janeiro (UFRJ) Rio de Janeiro Brazil; ^3^ The Oceanography Graduate Program of University of Rio de Janeiro State (PPG‐OCN/UERJ) Rio de Janeiro Brazil; ^4^ Department of Marine Biology Fluminense Federal University (UFF) Niterói Brazil; ^5^ Technological Federal University of Paraná – Campus Dois Vizinhos Dois Vizinhos Brazil; ^6^ AquaRio—Aquário Marinho do Rio de Janeiro Rio de Janeiro Brazil; ^7^ IMAM‐AquaRio–Rio de Janeiro Aquarium Research Center Rio de Janeiro Brazil

**Keywords:** 16S rRNA, aquaria, captivity, elasmobranch, estuary, Guanabara Bay

## Abstract

Bacterial communities of two critically endangered rays from the South Atlantic, the butterfly ray (*Gymnura altavela*) and the groovebelly ray (*Dasyatis hypostigma*), were described using 16S rRNA gene metabarcoding. The study characterized the bacterial communities associated with (i) *G. altavela* in natural (*in situ*) and aquarium (*ex situ*) settings, (ii) skin and stinger of *G. altavela*, and *D. hypostigma* in aquaria, and (iii) newborns and adults of *D. hypostigma*. The results revealed potentially antibiotic‐producing bacterial groups on the skin of rays from the natural environment, and some taxa with the potential to benefit ray health, mainly in rays from the natural environment, as well as possible pathogens to other animals, including fish and humans. Differences were observed between the *G. altavela* and *D. hypostigma* bacteria composition, as well as between the skin and stinger bacterial composition. The bacterial community associated with *D. hypostigma* changed with the age of the ray. The aquarium environment severely impacted the *G. altavela* bacteria composition, which changed from a complex bacterial community to one dominated almost exclusively by two taxa, *Oceanimonas* sp. and *Sediminibacterium* sp. on the skin and stinger, respectively.

## Graphical abstract

This article focused on the bacterial communities in two poorly studied and endangered ray species from the southern Atlantic: the butterfly ray (*Gymnura altavela*) and the groovebelly ray (*Dasyatis hypostigma*). This is the first study to describe the bacterial community associated with these species in natural environments and aquariums.

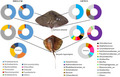

## 1 INTRODUCTION

Animals establish many relationships with microorganisms, which may be located superficially or in tissue microvilli (Rosenberg & Zilber‐Rosenberg, 2018). These microbiomes are important for the hosts, as they can facilitate nutrient absorption, regulate the host metabolism, and defend against pathogen invasion (Rosenberg & Zilber‐Rosenberg, 2018; Wilkins et al., 2019).

The significance of symbioses between marine teleost fishes and microbes has been examined in several studies (Chiarello et al., 2015; Givens et al., 2015; Larsen et al., 2013; Llewellyn et al., 2014; Roeselers et al., 2011). Similar studies of elasmobranchs (rays and sharks) are still scarce (Doane et al., 2017; Domingos et al., 2011; Givens et al., 2015; Ritchie et al., 2017) but have yielded interesting findings. For example, Ostrander and Keith (2005) demonstrated that although sharks in their natural environment often have open wounds, few skin infections have been documented. In these animals, wounds heal rapidly without exhibiting infection and maintain the normal function of the surrounding skin and a healthy microbiome (Ritchie et al., 2017). Elasmobranch skin, especially the epidermis and mucus, constitutes an active and dynamic immunological barrier, preventing pathogen colonization and subsequent infections that may lead to diseases. This is important during the reproduction period of many elasmobranchs since their copulation usually involves bites and dermal spine punctures on females, and rapid healing and absence of infection are required for continued female reproductive health. The skin mucus, secreted by epidermal cells and constituting a critical interface between the body surface and the surrounding water (Tsutsui et al., 2009), contains large numbers of microorganisms.

Rays are particularly known for a large number of accidents due to the use of their stingers, which have serrated edges and a very sharp tip, usually located at the base of the tail and used in defense. The group known as "stingrays" (Dasyatidae, Gymnuridae, Myliobatidae, and Urolopidae) possess a stinger covered with a tegumental sheath containing glandular venom cells, which can cause wound edema and necrosis (Barbaro et al., 2007; Haddad et al., 2012; Kalidasan et al., 2014). Half of the species in this group have lost this sheath, so the stinger causes only trauma and possible bacterial infections. The infection that develops following such wounds generally reflects the bacteria that are present on the body surface of the causative animal. Microorganisms that inhabit both the water and the skin of affected persons or animals can also be detected (Domingos et al., 2011; Haddad et al., 2004; Noonburg, 2005). Curiously, no studies have assessed whether the bacteria present on ray stingers are also the potential cause of these infections, although this is a possibility, as Buck et al. (1984) identified many bacteria pathogenic to humans in great white shark (*Carcharodon carcharias*) bites as the possible cause of subsequent infections.

With significant population declines and wide distributions, several species of rays and sharks are critically endangered (Dulvy et al., 2017). The spiny butterfly ray *Gymnura altavela* is classified as vulnerable and is critically endangered in the Southwest Atlantic (Vooren et al., 2007). This ray has a wide distribution, with records from the southwest, southeast, and northwest of the Atlantic Ocean and the Mediterranean and Black seas (Weigmann, 2016). In Brazil, it has been confirmed only off the south–southeast coast, where it faces strong fishing pressure and drastic declines in catches (Vooren et al., 2007), and is exposed to high levels of pollutants (Rosenfelder et al., 2012). Because of its vulnerability, studies are urgently required in all areas of knowledge and occurrence, especially in an important estuary such as Guanabara Bay in the state of Rio de Janeiro, Brazil, where this species is most abundant and uses the bay as a nursery ground (Gonçalves‐Silva & Vianna, 2018). This estuary is highly polluted and *G. altavela* is the main elasmobranch species in the area, drawing attention to potential characteristics that can aid the ray in tolerating this extreme environment.

The groovebelly ray (*Dasyatis hypostigma*), also referred to as the butter ray by fishers (Santos & Carvalho, 2004), is endemic to the South Atlantic; it is found on sand or mud bottoms in shallow coastal waters in southern Brazil and probably Uruguay and Argentina. This species is one of the most common rays accidentally caught by artisanal and commercial bottom trawlers and may also be negatively affected by habitat degradation and water pollution. It is little studied, and the International Union for Conservation of Nature (IUCN) lacks sufficient data to assess its conservation status, classifying it as data deficient (Charvet‐Almeida & Carvalho, 2006).

*G. altavela* and *D. hypostigma* are both threatened ray species, with significant declines in their populations. No studies on their associated microbiota are available. This is the first study to describe the bacterial community associated with these species, as well as the impact of aquarium settings on the ray bacteria composition. This study characterized the bacterial communities associated with (i) *G. altavela* rays in natural (*in situ*) and aquarium (*ex situ*) settings; (ii) *G. altavela* and *D. hypostigma* skin and stingers in aquarium settings; and (iii) *D. hypostigma* newborns and adults.

## 2 EXPERIMENTAL PROCEDURES

### 2.1 Study area and sampling procedures

Ray specimens were collected from Guanabara Bay (22°41′–22°03′S, 43°28′–43°01′W) in southeastern Brazil. The bay is a shallow coastal tropical estuarine ecosystem heavily impacted by direct discharge of untreated sewage, with many surrounding commercial activities including industries, shipyards, ports, an oil refinery, and oil and gas terminals (Kjerfve et al., 1997). Even with all this anthropic pressure, the bay still supports a significant and extremely diverse fish population (Prestelo & Viana, 2016; Silva‐Junior et al., 2016). Specimens of *Gymnura altavela* (Linnaeus, 1758) were sampled in an area with the highest occurrence rates (Silva‐Junior et al., 2016). This area is shallower, less saline, and more eutrophic than the rest of the bay and receives drainage from highly polluted rivers, with less water circulation compared with other areas of the estuary (Kjerfve et al., 1997; Silva‐Junior et al., 2016).

Samples were obtained from *G. altavela* and *D. hypostigma* rays living in an aquarium at AquaRio (Rio de Janeiro Marine Aquarium). The aquarium is located in the city of Rio de Janeiro, Brazil, and is the largest marine aquarium in South America, comprising 26,000 square meters of built area and 4.5 million liters of water. The microbiological material was obtained from rays held in quarantine tanks used to acclimatize fish in a controlled environment, free of disease‐causing microorganisms, for subsequent accommodation in the exhibition aquaria.

Three individual *G. altavela* rays were collected from Guanabara Bay, processed on the boat, and released alive in the bay (IBAMA/Brazilian Institute of Environment and Water Resources, Scientific Capture Permit No. 055, 05/05/2005). The rays were caught with a silicone mesh net and carefully removed from the water. Eight swabs were taken from each dorsum and stored in glass tubes containing 1 ml of 1× TE buffer (pH 8, 10 mM Tris + 1 mM EDTA). The stingers were cut with flared pliers and stored in an autoclaved plastic tube. Bottom‐water samples from the same capture site were collected with a Van Dorn bottle, and sediment was obtained with an Ekman grab. The material was stored at −20°C until processing upon arrival at the Microbial Molecular Ecology Laboratory (LEMM).

To compare the impact of an aquarium environment on the ray microbiome, we had access to a different group of marine aquarium specimens. Briefly, three specimens caught by artisanal fishers with gillnets off the coast of the state of Rio de Janeiro were acclimatized in an aquarium for about a month. After acclimatization, samples of mucus from the stinger and dorsal surface of these three *G. altavela* and *D. hypostigma* individuals were collected by a nonlethal method. Mucus samples were also collected from the dorsa of three newborn *D. hypostigma* rays born in captivity. The stingers could not be collected, because of their small size and the impossibility of cutting by the same microbiological sample collection method used for wild rays.

Due to logistical reasons and the locally endangered status of *G. altavela*, with low wild populations, *G. altavela* specimens were difficult to collect and few captive specimens were available. However, despite the relatively small sample size, we believe the data are important due to the size of the effect under study and the importance of assessments that can aid in conservation efforts for this species. This study followed the recommendations of the Guide for the Care and Use of Laboratory Animals of the Laboratory of Biology and Fisheries Technology, Institute of Biology, UFRJ, and was approved by the IMAM‐AquaRio (Aquarium Research Center) Committee on the Ethics of Animal Experiments.

### 2.2 Molecular methods

At the laboratory, the TE buffer from the swabs was concentrated using a vacuum centrifuge (SpeedVac), evaporating the solvent and allowing the DNA to be extracted from the buffer. The stingers were crushed to a fine powder in a porcelain mortar and pestle. For the environmental samples, 500 ml of seawater was filtered through 0.22‐μm filters (Millipore) and 2 g of sediment was used. Sample DNA (water, sediment, mucus, and stinger samples) was extracted using the PowerSoil® DNA Isolation Kit (Mo Bio Laboratories, Carlsbad, CA, USA), following the manufacturer's instructions. The DNA preparations were observed after electrophoresis in 1% agarose gel – 80 V in 1× TAE buffer (40 mM Tris, 20 mM Acetate, and 1 mM EDTA) for 1 hr to assess their integrity. The concentration of extracted DNA was determined using the Invitrogen™ Qubit® dsDNA High‐Sensitivity (HS) Assay Kit (Life Technologies) and stored at −80°C.

The 16S rRNA V4 variable region was amplified using 515 (5ʹ‐GTGYCAGCMGCCGCGGTAA‐3′)/806 (5ʹ‐GGACTACNVGGGTWTCTAAT‐3′) PCR primers (Caporaso et al., 2011) in a single‐step 30‐cycle PCR. Approximately 10 ng of extracted genomic DNA was used for the PCR amplification in a 20‐μl PCR reaction using the HotStarTaq Plus Master Mix Kit (Qiagen Ltd., USA). Each PCR reaction contained 13 μl dd H_2_O, 2 μl 10× buffer, 0.4 μl MgCl_2_, 0.4 μl dNTP at 200 μM (final concentration), 1 μl of each forward and reverse primer at 0.5 μM (final concentration), 0.12 μl HotStarTaq plus DNA polymerase, and 2 μl of each extracted DNA. PCR was carried out on a Mastercycler® pro S thermocycler (Eppendorf). The PCR conditions were as follows: 94°C for 3 min, followed by 28 cycles (5 cycles used on PCR products) at 94°C for 30 s, 53°C for 40 s, and 72°C for 1 min, and a final elongation step at 72°C for 5 min. Sequencing was performed at the Argonne Laboratory (http://ngs.igsb.anl.gov, Chicago) using an Illumina MiSeq platform, following the manufacturer's instructions.

### 2.3 Sequence analysis

The Quantitative Insights into Microbial Ecology—QIIME 2 (version 2017.10) (https://qiime2.org/) software package was used to process the raw sequence data (Bokulich et al., 2017). The reads were obtained after demultiplexing with q2‐DEMUX, with an average sequence length of 250 bp. The quality was filtered and replicated, and chimeras were removed using q2‐DADA2 (Callahan et al., 2016). Representative sequence sets for each DADA2 sequence variant were used for taxonomic classification. The remaining high‐quality sequences were grouped into operational taxonomic units (OTUs) with 99% sequence identity, using vSEARCH (Rognes et al., 2016). A representative sequence for each phylotype was aligned against the Greengenes database (Desantis et al., 2006), using PyNAST (Caporaso et al., 2011), and sequences were classified using the Greengenes taxonomy via the RDP classifier (Wang et al., 2007). Singletons, chloroplast plastids, mitochondria, and archaeal sequences were removed from the dataset before further analyses. For all OTU‐based analyses, the original OTU table was rarified to a depth of 1900 sequences per sample to minimize the effects of sampling effort on the analysis. The QIIME 2 package was also used to generate weighted UniFrac distance matrices (Lozupone et al., 2006) and α‐diversity metrics, including richness, diversity indexes, and rarefaction curves. All sequences were deposited in the NCBI Sequence Read Archive database, under Accession Number PRJNA484603.

### 2.4 Statistical analyses

For the β‐diversity analysis, the matrices from the sequencing data were ordered through non‐metric multidimensional (NMS) ordering (Kruskal, 1964; Mather, 1976) with the aid of the Past v.3.x (Paleontological Statistics) software package (Hammer et al., 2001). A permutational multivariate variance analysis (PERMANOVA) (Kelly et al., 2015) was applied, again using the Past v.3.x program to evaluate variations between the different sample types (Hammer et al., 2001).

The α‐diversity was calculated with QIIME, using the observed Chao1 (Chao, 1984) and OTU metrics. To evaluate variations between different sample types, a parametric analysis of variance (ANOVA) was used for normal data, while the nonparametric Kruskal–Wallis test was performed for non‐normal data. All results were considered significant at *p* < 0.05. Venn diagram analyses were carried out using the InteractiVenn tool, to determine the number of exclusive and shared OTUs of the abiotic and biotic samples (Heberle et al., 2015). Similarity percentages (SIMPER) were used to determine the relative contributions of OTUs to the observed similarity/dissimilarity within each sample type (Clarke & Warwick, 2001).

## 3 RESULTS

### 3.1 Bacterial community composition of dorsal skin and stinger of *G. altavela* from natural and aquarium environments

**FIGURE A1:**
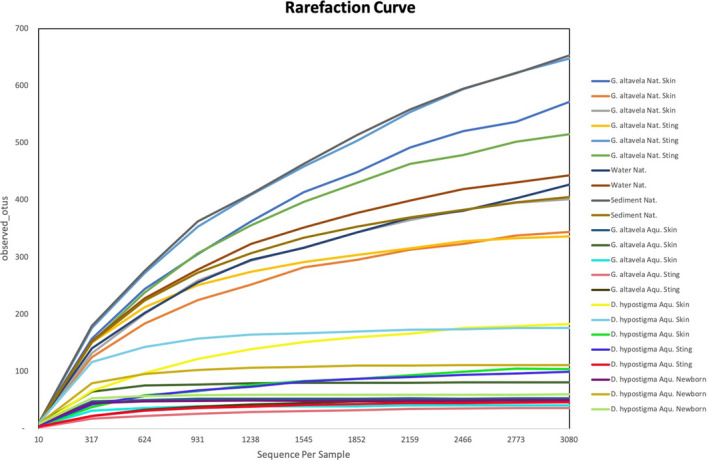
Rarefaction curves showing the diversity detected compared to the predicted total diversity

The logarithmic rarefaction curves obtained for the sequence data indicated that most of the species occurring in the samples were detected, and also showed that the number of OTUs in the samples from the natural environment was consistently higher than in the samples from aquaria when the same number of sequences was abstracted (Figure A1).

**FIGURE 1:**
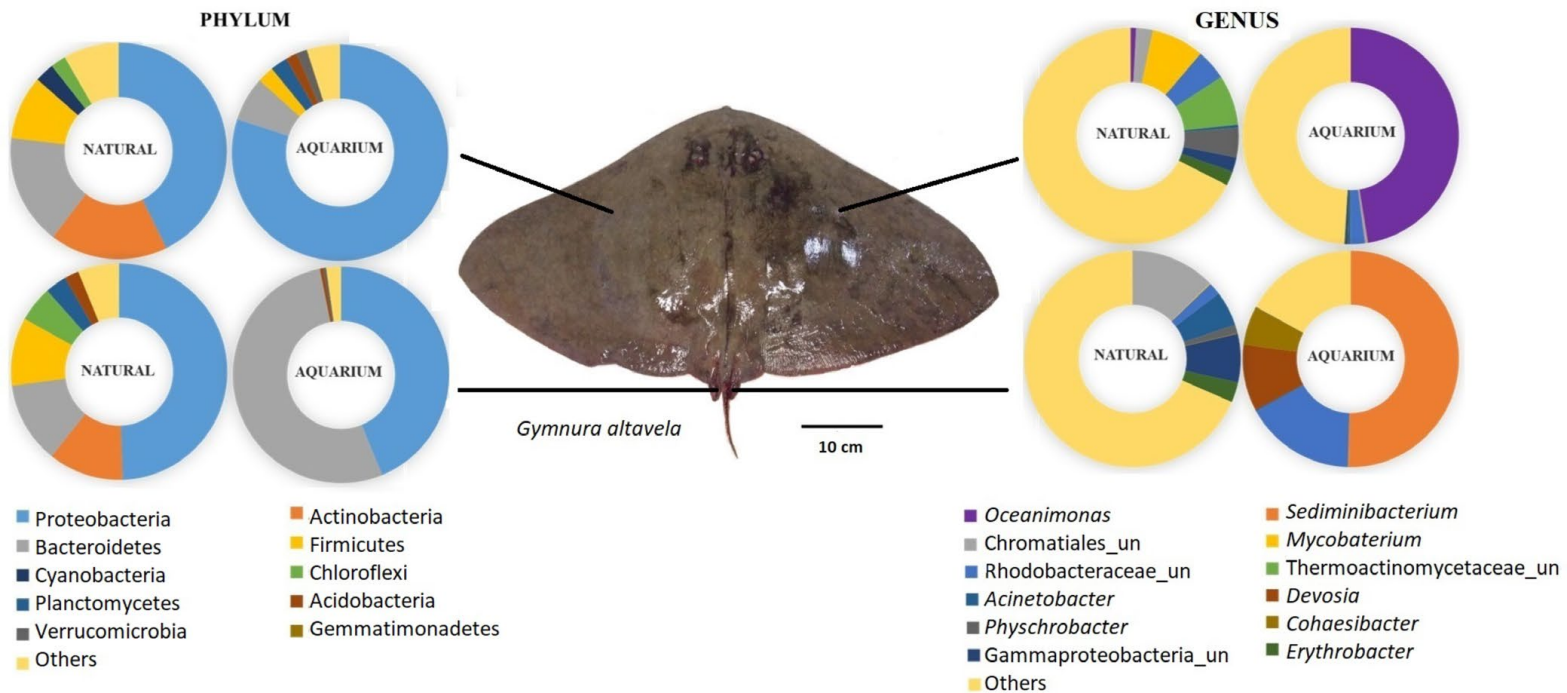
Relative OTU abundance from the 16S rRNA gene sequencing data (Illumina MiSeq Series) at the phylum and genus levels in stinger and skin samples from *Gymnura altavela* rays in the natural and aquarium environments

**FIGURE A2:**
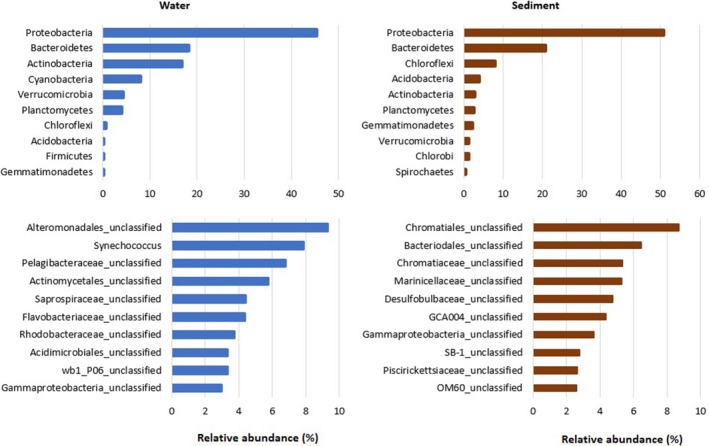
Relative abundances of dominant phyla from the natural environment—Guanabara Bay water and sediment samples

The composition of bacteria in the dorsal skin and stinger of *G. altavela* differed in rays from the natural and aquarium environments (Figure 1). The dominant phyla on the skin and the stinger of rays from the natural environment were the same, Proteobacteria, Bacteroidetes, and Actinobacteria (Figure 1). In individuals from the aquarium, however, the skin was dominated almost exclusively by Proteobacteria (≈80%), and the stinger by Bacteroidetes (≈53%) and Proteobacteria (≈44%). An increase in Proteobacteria and Bacteroidetes was noted for the skin and sting of rays from the aquarium, respectively, while no significant difference in Actinobacteria was found. This indicates a greater OTU distribution from different phyla in the wild specimens. The dominant phyla on the skin and stinger from the natural environment were the same as in the Guanabara Bay water and sediment samples (Figure A2).

The difference between the natural and aquarium environment microbiomes was high at the genus level. In rays from the bay, *Mycobacterium* was most abundant on the skin, followed by a genus of the family Thermoactinomycetaceae and by *Psychrobacter*. On stingers, the most abundant genera were from the order Chromatiales, the class Gammaproteobacteria, and *Acinetobacter* (Figure 1). In aquarium rays, bacteria of the skin and stinger were largely dominated by only one genus, *Oceanimonas* in the skin samples (47.5%) and *Sediminibacterium* in the stinger samples (50%). Relative abundances of members of the family Rhodobacteraceae and *Devosia* were detected (Figure 1). The most abundant genus in Guanabara Bay sediment, a member of Chromatiales, was also the most abundant on ray stingers and was among the ten most abundant genera on the skin of rays from the bay (Figure A2).

The Similarity percentage analysis (SIMPER) highlighted the OTUs that were primarily responsible for the differences between sample types, using the relative OTU abundances in each sample. Comparing *D. altavela* skin and stingers from the natural and aquarium environments, the 10 most numerous OTUs comprised 35.67% of the dissimilarity among the samples (Table 1). The SIMPER analysis indicated that *Sediminibacterium* and *Bacillus* were the main contributors to the dissimilarity of stingers from aquarium rays, and *Oceanimonas* was the main contributor to the skin from aquarium rays, while the other eight taxa were the main contributors to the natural environment. In rays from the bay, the main contributor to skin similarity was *Mycobacterium*, while a genus of order Chromatiales contributed most to the similarity of stingers.

**Table 1:** Identification and ranking of OTU contributions by SIMPER analysis on bacterial community structure among all sample types of all habitats

Taxon	Av. Dissim.	Contrib. %	Cum. %	Mean		
Impact of aquaria settings				*G. altavela* Aquarium—skin	*G. altavela* Aquarium—stinger	*G. altavela* Natural—skin
*Sediminibacterium*	12.74	13.58	13.58	0	14,900	0
*Oceanimonas*	4.19	4.47	18.05	3610	13.5	380
*Bacillus*	3.15	3.36	21.41	90.3	4050	0
*Mycobacterium*	2.90	3.09	24.50	0	0	4060
*(o) Chromatiales*	2.75	2.93	27.43	0	0	671
*(f) Thermoactinomycetaceae*	2.21	2.35	29.78	0	0	1910
*(f) Geodermatophilaceae*	1.88	2.00	31.79	0	0	2120
*Geobacillus*	1.32	1.41	33.19	0	0	77.3
*Erythrobacter*	1.27	1.36	34.55	0	0	1240
*Geobacillus*	1.05	1.12	35.67	0	0	42

*G. altavela* × *D. hypostigma*				*G. altavela* Aquarium—skin	*G. altavela* Aquarium—stinger	*D. hypostigma* Aquarium—skin
*Sediminibacterium_*	24.32	27.35	27.35	0	14,900	0
*Oceanimonas*	16.81	18.90	46.25	3610	13.5	11,600
*Bacillus*	3.99	4.49	50.73	90.3	4050	0
*Oceanimonas*	2.42	2.72	53.45	506	0	1670
*(f) Rhodobacteraceae*	2.39	2.69	56.15	0	0	0
*Devosia_*	2.32	2.61	58.75	0	0	0
*Cohaesibacter*	1.53	1.72	60.48	0	0	0
*Rhodobacteraceae*	1.39	1.57	62.04	0	0	0
*Ochrobactrum*	1.24	1.39	63.43	688	16	165
*Staphylococcus*	0.95	1.07	64.50	535	324	209

Age‐related change *D. hypostigma*				*D. hypostigma* Adult—skin	*D. hypostigma* Adult—stinger	*D. hypostigma* Newborn—skin
*Oceanimonas*	19.23	19.65	19.65	11,600	10.5	0
*Sediminibacterium*	17.69	18.07	37.72	0	13,500	0
*Rhodobacteraceae*	3.299	3.37	41.09	0	2520	0
*Devosia*	3.197	3.266	44.35	0	2450	0
*Oceanimonas*	2.78	2.84	47.19	1670	0	0
*Cohaesibacter*	2.11	2.156	49.35	0	1620	0
*Rhodobacteraceae*	1.918	1.959	51.31	0	1470	0
*Corynebacterium*	1.58	1.614	52.92	0	24.5	802
*Staphylococcus*	1.271	1.298	54.22	209	10.5	729
*Halomonas*	1.085	1.109	55.33	216	618	0

The multidimensional scaling (MDS) (beta diversity analysis) of *G. altavela* skin and stingers from the natural and aquarium environments indicate a clear difference in the bacterial communities between the two environments. The difference between skin and stinger was higher for rays from the aquarium than from the bay (Figure 3a).

The Venn Diagram showed that only a few OTUs were shared between rays from the two environments (Figure 4a). Only three OTUs were shared among all samples. The vast majority of OTUs belonged to samples from the natural environment, and a large number of OTUs were exclusive to each type of sample. However, approximately half of the OTUs associated with the stingers of rays from the natural environment were also shared with the skin of the same rays. A high number of exclusive OTUs were observed on the skin samples from captive rays compared with the OTUs of the stinger samples, revealing a change in the skin bacteria and the loss of a bacterial community from stingers of these rays. These results suggest a high impact of captivity on the ray bacterial community.

**FIGURE A3:**
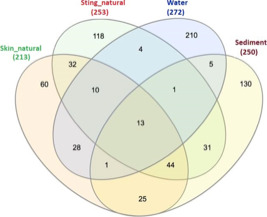
Venn diagram of skin samples from *Gymnura altavela* rays in the natural environment, Guanabara Bay water, and sediment

Concerning the OTU numbers and Chao index, wild rays showed higher bacterial diversity, similar to the water and sediment samples, while aquarium samples showed lower bacterial diversity. The aquarium ray samples were significantly different from wild rays (Kruskal–Wallis, *p* < 0.05) (Table 2). A total of 100 OTUs were shared exclusively between *G. altavela* rays from the bay and the bay sediment, higher than the number of exclusive OTUs shared between the rays and the water (42 OTUs), indicating a stronger influence of the sediment on the ray bacterial community (Figure A3).

**Table 2:** Diversity indices for the *Gymnura altavela* and *Dasyatis hypostigma*: skin, stinger, newborn, natural, and aquarium environments

Sampling site	Species	Sample type	Observed OTUs	Chao index
Natural environmental	*G. altavela*	Skin	440.7 ± 117.4	561.9 ± 235.1
		Stinger	502.3 ± 158.0	637.0 ± 296.4
		Water	434.7 ± 21.3	581.1 ± 23.5
		Sediment	528.7 ± 174.2	693.3 ± 323.8
Aquarium	*G. altavela*	Skin	155.2 ± 43.8	173.1 ± 46.5
		Stinger	72.8 ± 38.5	84.4 ± 52.8
Aquarium	*D. hypostigma*	Skin	58.1 ± 20.8	58.2 ± 20.9
		Stinger	43.5 ± 8.6	48.0 ± 8.5
		Skin—newborn	73.9 ± 33.1	74.4 ± 33.3

### 3.2 Bacteria associated with *G. altavela* and *D. hypostigma* skin and sting in aquarium settings

**FIGURE 2:**
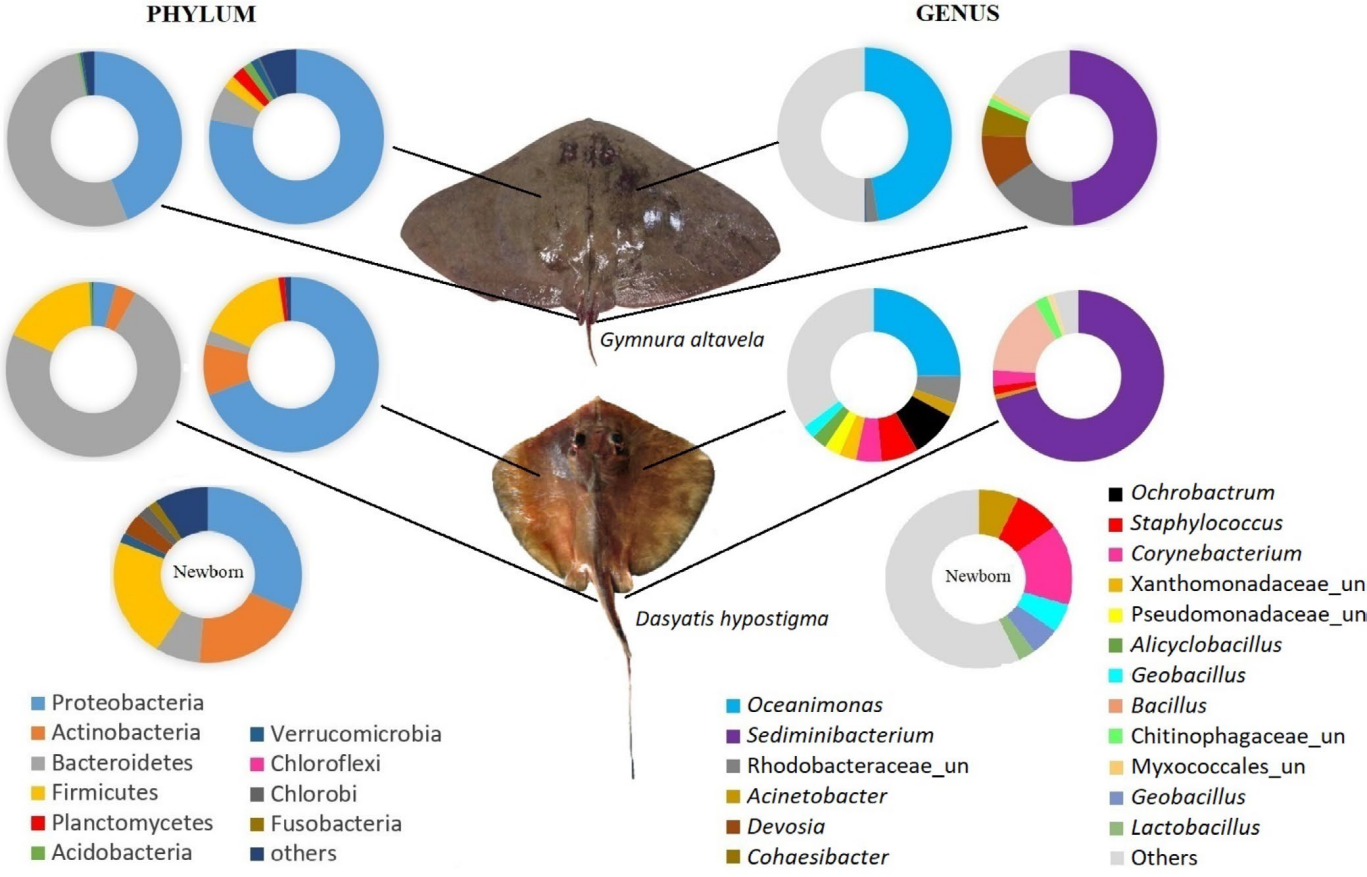
Relative OTU abundance from the 16S rRNA gene sequencing data (Illumina MiSeq Series) at the phylum and genus levels of stinger and skin samples from *Gymnura altavela* and *Dasyatis hypostigma* (newborn and adult) rays in aquarium settings

The most abundant phyla on *G. altavela* and *D. hypostigma* in aquarium settings were the same on the skin and stingers, Proteobacteria and Bacteroidetes, respectively (Figure 2). Firmicutes were more abundant on the skin and stinger of *D. hypostigma* than *G. altavela*.

The distribution of the most abundant genera was similar to that for phyla. The most abundant genera on both ray species were the same: *Oceanimonas* on the skin and *Sediminibacterium* on the stinger (Figure 2). The second most abundant genera on the stinger were a member of Rhodobacteraceae in *G. altavela* and *Bacillus* in *D. hypostigma*.

The SIMPER analysis of the two species in aquarium settings indicated that *Sediminibacterium* and *Oceanimonas* were the main contributors to the stinger and skin dissimilarity between the two species, respectively (Table 1). A genus of Rhodobacteraceae and the genera *Devosia* and *Cohaesibacter* also contributed to stinger dissimilarity in *D. hypostigma*, while *Bacillus* contributed to stinger dissimilarity in *G. altavela*.

**FIGURE 3:**
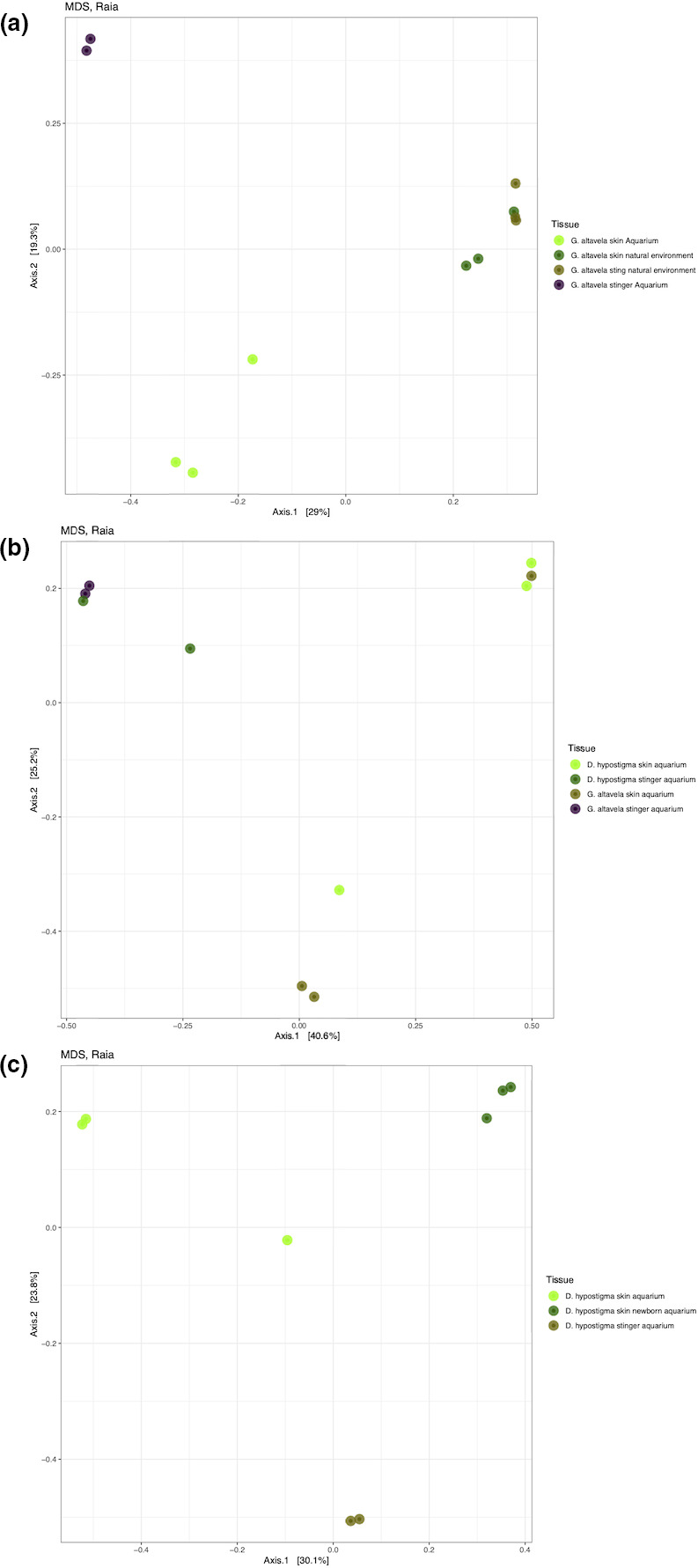
The multidimensional scaling analysis (MDS): (a) samples of stinger and skin from *Gymnura altavela* rays in natural and aquarium environments; (b) samples of stinger and skin from *Gymnura altavela* and *Dasyatis hypostigma* from aquaria; and (c) samples from adult and newborn *D. hypostigma* rays from aquaria

The MDS of the skin and stingers of both rays in aquarium settings indicated that in both species, the bacterial communities of the stinger and skin were distinct. The stinger bacterial communities of the two species were very similar, while the skin bacterial communities were different, except for one individual of *D. hypostigma* and one *G. altavela* (Figure 3b).

**FIGURE 4:**
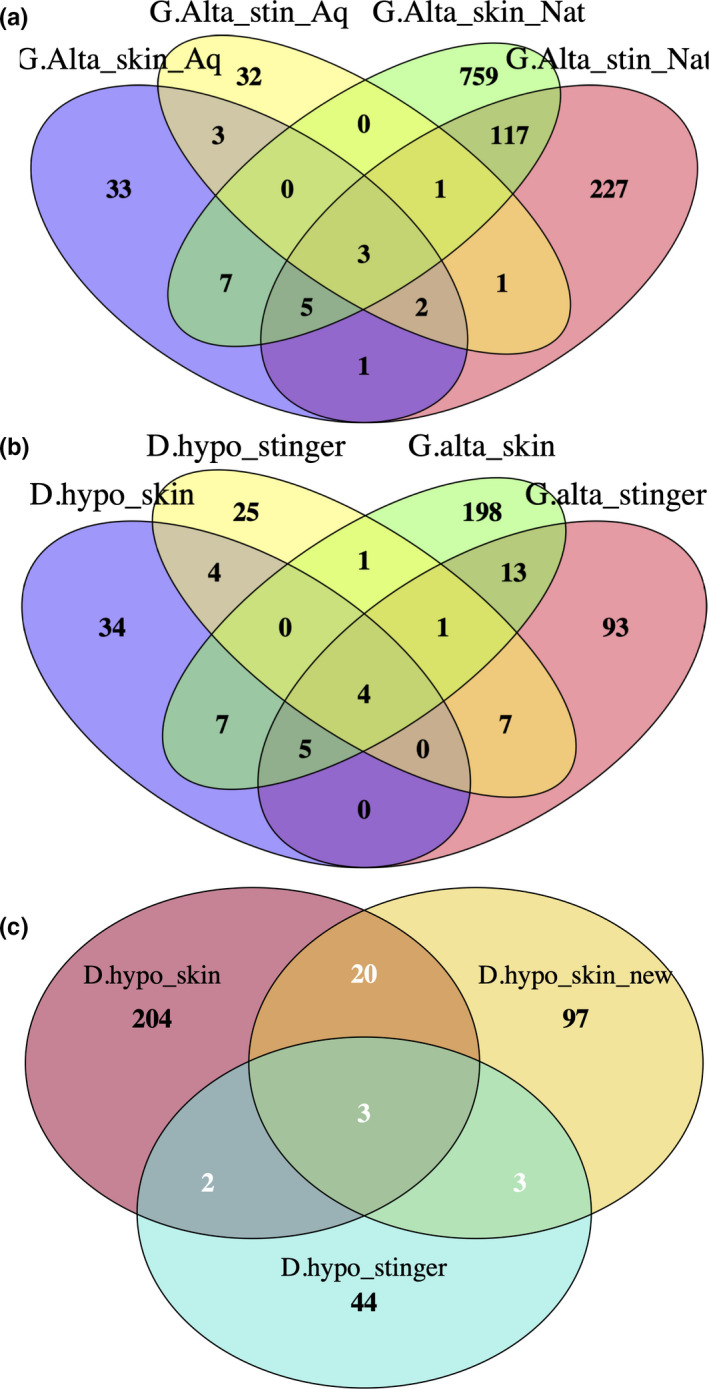
Venn diagram of (a) samples of stinger and skin from *Gymnura altavela* rays in natural and aquarium environments; (b) samples of *G. altavela* and *Dasyatis hypostigma* stingers and skin from aquaria; and (c) samples of adults and newborns of *D. hypostigma* from aquaria

The Venn Diagram for the two species in the aquarium revealed a low number of shared OTUs. Although the most abundant bacterial genera on the stinger and skin of the two species were the same, other bacterial community members were different (Figure 4b).

### 3.3 Age‐related bacterial community changes in *D. hypostigma* in aquarium settings

The three most abundant bacterial phyla associated with the skin of newborn and adult *D. hypostigma* rays in the aquarium were the same, Proteobacteria, Firmicutes, and Actinobacteria. Nevertheless, the genera distributions were different. A predominance of *Oceanimonas* (25.8%) was observed in adults, similarly to *G. altavela*. The most abundant genus on newborn *D. hypostigma* individuals was *Corynebacterium*, followed by *Staphylococcus* and a genus belonging to the family Xanthomonadaceae. These genera were less abundant in adult *D. hypostigma* individuals (Figure 2).

The SIMPER analysis of the adult and newborn *D. hypostigma* rays in the aquarium indicated that in adults, *Sediminibacterium*, followed by Rhodobacteraceae, *Devosia*, and *Cohaesibacter* were the main contributors to stinger dissimilarity (Table 1), while *Oceanimonas* was the main contributor to skin dissimilarity. In newborns, the main contributors to dissimilarity were *Corynebacterium* and *Staphylococcus*.

The MDS of newborn and adult *D. hypostigma* rays in the aquarium indicated large differences in the bacterial community associated with the three types of samples, adult and newborn skin and adult stingers (Figure 3c).

Although the Venn Diagram of adults and newborn rays of *D. hypostigma* in the aquarium indicated a large number of OTUs unique to each type of sample, the largest number of shared OTUs (20) was detected in skin samples from adult and newborn rays, and are presumably members of the bacterial community associated with *D. hypostigma* individuals regardless of age (Figure 4c).

## 4 DISCUSSION

In recent years, the microbiomes of fish skin have been increasingly studied, and it is becoming clear that microorganisms associated with fish play an important role in the health of these animals (Krotman et al., 2020). Studies on animal microbiomes are important for understanding the invasion of non‐native species and responses to pathogens and built environments such as aquaria (McFall‐Ngai et al., 2013; Wilkins et al., 2019). This study described the bacterial community associated with *G. altavela* rays. It also revealed the impact of an aquarium environment on bacteria of this species in comparison with another ray species, *D. hypostigma*, as well as age‐related changes in the *D. hypostigma* bacterial composition. Due to the endangered status of the specimens, we employed a non‐destructive sampling procedure, swabbing the captured rays *in situ* and immediately releasing them.

### 4.1 The impact of the natural environment and aquarium settings on bacteria from *G. altavela*

Analyses using non‐metric multidimensional scaling (NMDS), Venn diagram, and the relative abundance of bacterial groups demonstrated differences in bacteria between *G. altavela* individuals and abiotic samples (water and sediment) from the bay. These *G. altavela* rays have specific bacteria, which may establish a symbiosis to benefit the health of the species. The greater similarity between the sediment and ray samples indicated that the sediment influenced the ray bacterial community more than the water. Several studies of aquatic animals have reported that host microbiomes are influenced by the presence of bacteria in the surrounding environment but that the hosts select this microbial community, and usually, the water has only a secondary influence (Chiarello et al., 2015; Doane et al., 2017; Kearns et al., 2017; Kueneman et al., 2014; Larsen et al., 2013; Santos et al., 2014).

The most abundant phyla of bacteria from *G. altavela* in the natural environment were Proteobacteria, Bacteroidetes, Actinobacteria, and Firmicutes. The lack of previous studies on the *G. altavela* bacterial composition prevents a taxonomic comparison. However, genera belonging to Proteobacteria, Actinobacteria, and Firmicutes were the most abundant in the skin mucus of the Atlantic stingray *Dasyatis sabina* (Ritchie et al., 2017). These phyla were also reported as predominant in several other fish studies (Doane et al., 2017; Kearns et al., 2017; Ostrander & Keith, 2005).

Stingers of *G. altavela* from the bay showed higher richness indices and OTU numbers. This may indicate the importance of these complex bacterial communities associated with ray stingers. For instance, taxa such as the genus *Acinetobacter* and the family Piscirickettsiaceae, which can cause diseases in humans and other animals, including fish (Fryer & Hedrick, 2003; Li et al., 2017; Peleg et al., 2008; Wong et al., 2017), were detected on stingers of rays from the bay. To our knowledge, the bacterial community of fish stingers has not previously been investigated, unlike the venom in the stinger gland, which has been thoroughly studied regarding its characteristics, consequences after venom inoculation, and different treatments (Barbaro et al., 2007; Haddad et al., 2004, 2013; Kimura et al., 2014; Pedroso et al., 2007). Baldinger (1999), for example, described a serious injury caused by infection by *Pseudomonas aeruginosa*. These types of infections are caused by bacteria from the skin of the wound‐causing animal itself, the skin of the injured person, or the surrounding water (Kalidasan et al., 2014; Lim & Kumarasinghe, 2007; Murphey et al., 1992).

The high number of taxonomic groups and OTUs shared between the rays and the sediment reveals the influence of sediment on the ray bacterial community. Studies do not usually analyze the substrate, but for *G. altavela*, substrate bacteria assessments are essential, as this species is benthic (Froese & Pauly, 2018) and at times buries itself in the bottom sediments, which can facilitate microorganism colonization (Hameed et al., 2014; Marchant et al., 2002; Normand et al., 2014; Pfeffer et al., 2012).

The dominant bacterial order on the skin of rays from the bay was Actinomycetales, which is highly important to animals since some members of this taxon may be associated with protective mechanisms. Most antibiotics now originate from this bacterial group. Furthermore, other organisms maintain a protective symbiosis with Actinobacteria, which increases the defensive capacity of the holobiont (Flórez et al., 2015; Kaltenpoth, 2009). Other antibiotic‐producing groups found in abundance on the skin of rays from the bay were *Kocuria*, *Psychrobacter*, and *Lysobacter* (Martín et al., 2013; Palomo et al., 2013; Panthee et al., 2016; Ritchie et al., 2017; Uzair et al., 2008). Antibiotic‐producing bacteria belonging to *Psychrobacter* have been previously isolated from another ray species, *Dasyatis sabina* (Ritchie et al., 2017). Species of *Lysobacter* produce peptides that damage the cell walls or membranes of other microbes. This genus is regarded as a rich source for novel antibiotics, including a new group of antibiotics, Katanosins (also known as lysobactins) (Panthee et al., 2016). *Kocuria* antibiotic producers have been isolated from other marine organisms, such as sponges (Palomo et al., 2013) and algae (Martín et al., 2013). The antibiotics produced by *Kocuria* include a new antibiotic (Kocurin), which is effective against methicillin‐resistant *Staphylococcus aureus* (MRSA). *Kocuria* was the main contributor to the dissimilarity between samples from rays in the bay, compared with sediment and water samples. This indicates the significant importance of this genus to the bacterial community of *G. altavela* rays from the bay.

The most abundant genus in skin samples from rays in the bay was *Mycobacterium*. This is one of the major zoonotic bacterial pathogen groups and includes several disease‐causing species that can affect a wide variety of marine and freshwater fishes (Eddyani et al., 2004; Gauthier, 2015; Gauthier & Rhodes, 2009; Lewis et al., 2003; Ucko et al., 2002). Transfer of these bacteria to humans may also cause diseases, such as the rare disease known as aquarium granuloma, which typically affects individuals who work with fish or keep home aquariums (Huminer et al., 1986). This result also reveals the possible impact of a eutrophic environment, such as Guanabara Bay, on the microbiome of marine organisms and the risk of handling and consuming rays.

The incredible ability of elasmobranchs to quickly recover from wounds without infection has increased interest in identifying antimicrobial compounds from these animals. Bacteria compete intensely, both intra‐ or inter‐specifically, for space and resources, and are therefore expected to have developed mechanisms to prevent the colonization of other bacteria (Falagas et al., 2008). The ray dermal mucus has been studied in an attempt to explain the absence of elasmobranch infections (Conceição et al., 2012; Domingos et al., 2011; Ritchie et al., 2017; Vennila et al., 2011). The results of the present study reveal a possible role of bacteria from the ray in protecting the host.

Other important taxa found on ray skin were the families Rhodobacteraceae and Thermoactinomycetaceae. Rhodobacteraceae can be involved in promoting the initial formation of biofilm, that is, adhering to surfaces (Elifantz et al., 2013; Kviatkovski & Minz, 2015), and as a eukaryote mutualist (Simon et al., 2019). The presence of Thermoactinomycetaceae, the second most abundant family in skin samples, is interesting since these bacteria grow only at high temperatures, and their presence in marine environments could only occur through terrestrial discharges (Pathom‐Aree et al., 2006).

### 4.2 Comparison of bacteria from *G. altavela* and *D. hypostigma* skin and sting in aquarium settings

Public aquariums open to visitors, such as the Rio de Janeiro AquaRio, are important for environmental education, tourism, and entertainment. Despite their popularity and the implications that microbial community structure can have for both host health and habitat function, little is known about the impact of the aquarium environment on the microbial community associated with marine organisms, especially in rays (Kearns et al., 2017).

Our data revealed a significant change in bacterial composition and a decrease in beta diversity indices for the captive ray. The impact of these changes on the animals is still not clear, although it is known that impacts on the microbial community can alter the health of the animal host (Simon et al., 2019). This may increase the susceptibility of rays to diseases and reduce the defense capacity of the stingers. Furthermore, it is essential to maintain the health of animals housed in large aquaria, since one individual with disease symptoms may spread the disease to several others, placing all animals at risk. These ailments can range from skin diseases to more serious internal diseases caused by both viruses and bacteria (Bernoth & Crane, 1995; Ferguson et al., 1994; Puk et al., 2017). Also, some bacteria responsible for fish diseases can be transmitted to humans (Gauthier, 2015), with fishers as the main victims (Haddad et al., 2013).

The captive *G. altavela* and *D. hypostigma* showed less‐complex bacterial communities, with those of skin and stingers essentially dominated by single taxa. The bacterial community associated with skin was dominated almost exclusively by *Oceanimonas*, a potential pathogen. Some virulence genes have been identified in this species (Yeganeh et al., 2015), and some species of this genus have been previously isolated from allantoin‐rich seawater (Numata & Morisaki, 2015). Allantoin is produced from uric acid, and elasmobranchs contain unusually high concentrations of uric acid in their body fluids (Schooler et al., 1966). This can explain the very high abundance of these potentially pathogenic taxa on ray skin. The high abundance of *Oceanimonas* may occur due to decreases in the natural protection afforded by the bacterial community of ray skin, such as loss of the antibiotic‐producing bacteria *Kocuria*, *Lysobacter*, and *Psychrobacter*. It may be interesting that the potentially pathogenic *Mycobacterium* was absent from the skin of rays in aquaria. Further research is required to explain this difference between the wild and aquarium rays.

Similar to the skin of aquarium rays, the stingers were dominated almost exclusively by a single genus, *Sediminibacterium*. These taxa are usually found in microbial aggregates, such as flocs, granules, and biofilms isolated from aquaria, reservoirs, and freshwater (Ayarza et al., 2015; Kang et al., 2014; Kim et al., 2016; Pinto et al., 2018; Qu & Yuan, 2008). The predominance of only one bacteria genus associated with the skin or stinger of both ray species in aquarium settings, which was not seen in rays from the bay, might be due to aquarium impacts.

### 4.3 Bacterial composition changed with the age of *D. hypostigma* in the aquarium setting

The skin of newborn *D. hypostigma* in aquaria exhibited the same predominant phylum as adults, although with different relative abundances of genera. These changes in genera may be related to age. *Corynebacterium* and *Staphylococcus* were the main contributors to the dissimilarity of newborn rays and adults; members of both genera are potentially pathogenic to animals, including humans. As mentioned above, *Kocuria* can produce antibiotics that have an antagonist effect on *S. aureus* growth. Therefore, the absence of *Kocuria* from newborn rays in aquarium settings may explain the high abundance of *Staphylococcus* in the microbial community of these animals. These results suggest that newborn rays have a larger number of potential pathogens, which reinforces the importance of careful maintenance of this age group.

## 5 CONCLUSIONS

The butterfly ray (*Gymnura altavela*) and the groovebelly (or butter) ray (*Dasyatis hypostigma*) are little studied and locally endangered species, with significant declines in their populations in the South Atlantic. No previous studies on their associated bacteria were available.

Despite the small sample size, our high‐throughput sequencing data revealed important information about the bacterial community associated with these species, as well as the impact of aquarium settings on the bacterial community from the ray. Together, our findings demonstrated that the bacterial community of rays from the natural environment is complex, with a high diversity of taxa, some of which may establish symbiotic associations to benefit the health of these animals, and/or may cause diseases in humans and other animals, including fish. This study also revealed the impact of an aquarium environment on the diversity and richness of the ray bacteria, as the stinger and skin bacterial communities were dominated basically by a single genus. Further studies are required to understand the functional relationships between the microorganisms and rays (adults and newborns), especially concerning *Oceanimonas* and *Sediminibacterium*, the dominant bacterial taxa in the captive rays.

## CONFLICT OF INTEREST

None declared.

## AUTHOR CONTRIBUTIONS

**Fernanda Gonçalves e Silva:** Conceptualization (equal); data curation (equal); formal analysis (equal); investigation (lead); methodology (equal); writing‐original draft (lead); writing‐review & editing (equal). **Henrique Fragoso dos Santos:** Data curation (equal); formal analysis (equal); investigation (equal); writing‐original draft (equal); writing‐review & editing (equal). **Deborah Catharine de Assis Leite:** Investigation (equal); methodology (equal). **Daniela Silva Lutfi:** Resources (equal); validation (equal). **Marcelo Vianna:** Conceptualization (equal); funding acquisition (equal); resources (equal); supervision (equal); writing‐original draft (equal); writing‐review & editing (equal). **Alexandre Soares Rosado:** Conceptualization (equal); funding acquisition (equal); project administration (equal); resources (equal); supervision (equal); writing‐original draft (equal); writing‐review & editing (equal).

## ETHICS STATEMENT

This study followed the recommendations of the Guide for the Care and Use of Laboratory Animals of the Laboratory of Biology and Fisheries Technology, Institute of Biology, UFRJ, and was approved by the IMAM‐AquaRio (Aquarium Research Center) Committee on the Ethics of Animal Experiments. This study was conducted under a permit issued by IBAMA/Brazilian Institute of Environment and Water Resources, Scientific Capture Permit No. 055, 05/05/2005.

## Data Availability

All data generated or analyzed during this study are included in this published article apart from raw sequence reads that were uploaded to the National Center for Biotechnology Information's Sequence Read Archive under the accession number PRJNA484603: https://www.ncbi.nlm.nih.gov/bioproject/PRJNA484603.
